# The influence of the Youth Olympic Games on the well-being of youth athletes

**DOI:** 10.3389/fspor.2025.1662936

**Published:** 2025-10-29

**Authors:** Jannicke Stålstrøm, Marina Iskhakova, Erik P. Andersson

**Affiliations:** ^1^ School of Sport Sciences, Faculty of Health Sciences, UiT The Arctic University of Norway, Tromsø, Norway; ^2^ Faculty of Education and Social Work, The University of Sydney, Sydney, NSW, Australia; ^3^ Research School of Economics, Australia National University, Canberra, ACT, Australia; ^4^ Swedish Winter Sports Research Centre, Department of Health Sciences, Mid Sweden University, Östersund, Sweden

**Keywords:** athlete development, elite youth sport, mental health, sport psychology, sport participation, mixed-methods research

## Abstract

**Introduction:**

Participating in sports is more than a competition; it is an avenue for personal growth and development, especially for young athletes. The Youth Olympic Games (YOG), established by the International Olympic Committee in 2010, is a unique platform for athletes aged 15–18 to showcase their skills while gaining invaluable life experiences. This study explores the influence of participation in the YOG, focusing on athletes' physical, mental, social, and emotional well-being.

**Methods:**

Using a retrospective mixed-methods framework, 173 participants (47% female and 53% male) who competed in one of the four YOG events held between 2010 and 2016 were surveyed in 2017, followed by interviews 6 months later in 2018 with 30 of the participants. Quantitative data on 18 well-being items assessed on a 5-point Likert scale was analyzed with a one-sample Wilcoxon signed-rank test, and the interview data was analyzed using a top-down thematic approach.

**Results and discussion:**

Participants' responses were significantly above “neutral” (*P* < 0.001) on the 18 items about the YOG impact, suggesting a positive effect of the YOG across the four dimensions of well-being. The interviews complemented and informed the survey by providing deeper insights and context, to show that taking part in the YOG and its educational programs influence, and are important for the young elite athletes' psychometric development. Recognizing this impact, more attention should be given to developing tools and strategies to support the social and emotional well-being of youth elite athletes in sports. A better understanding of the impacts of participating in the YOG can foster a healthier, more informed generation of athletes and community members.

## Introduction

Sports participation and physical activity are known to have a positive influence on well-being and health in participants, and among youth (1–8). But elite sport participation can also harm and undermine an athlete's well-being (9–13) through internal and external pressure to succeed, through burnout, and through mental and physical injuries and/or disorders (14–16, 66). To succeed at the highest level sports performers in a competitive environment must focus on their holistic health (17).

As such, e.g., Giles et al. (11) developed a model that views athletes as people whose physical, mental, and social health are reflected in their well-being and ill-being. Therefore, athletes' holistic health is an integral aspect of who they are, both as sports performers and as people. Giles et al. (11) state that well-being in sport refers to the holistic state of an athlete's physical, mental, emotional, and social health, which enables them to thrive both within and beyond their sporting environment. Well-being in sport is often viewed as a dynamic process, influenced by factors such as training demands, competition pressures, recovery practices, and youth athletes' personal and social environment. For instance, Lundqvist (18) highlights that well-being in sport is not only about achieving peak performance but also about fostering a balanced and fulfilling life for athletes. This perspective aligns with the growing recognition that youth athletes' mental health and overall well-being are critical for their success and longevity in Olympic sport (19). Despite the lack of a universally agreed definition of well-being, it has both hedonic (subjective and emotional well-being) and eudaimonic components (psychological well-being) (18, 20). While hedonic and eudaimonic well-being are distinct, they are not mutually exclusive (18). Athletes often experience both forms of well-being simultaneously. For instance, the joy of winning a game (hedonic) can coexist with the sense of accomplishment from years of hard work and personal growth (eudaimonic) (21). Understanding and promoting both dimensions of well-being is essential for creating a supportive environment that nurtures athletes holistically.

In 2010, The International Olympic Committee aimed to address the youth athlete's health and well-being by establishing the Youth Olympic Games. Which was not only a platform for athletic competition but also an environment that shapes the holistic development of young elite athletes. The events uniqueness with combination of competition, education, and cultural exchange makes it an invaluable setting for studying youth elite athletes. Each YOG includes a “Learn and Share” or culture and education program, which reflects the Olympic philosophy of balancing body, will, and mind. These programs promote health and well-being, sportsmanship, and social values while addressing athlete-specific health issues and long-term habits, such as injury prevention, physical activity, motor skills, nutrition, anti-doping, and mental training (22). Included in these programs are adult Olympians participating as athlete role models, sharing their experiences and contributing to young athletes' sport–life balance (23).

It is the educational aspects that are part of YOG which make the connection between the Giles et al. model and the YOG potentially highly significant. Their model includes psychometric factors in a holistic framework for understanding the four well-being domains in sport: physical, mental, social, and emotional. Physical well-being refers to the physical health and fitness of an individual, including factors such as physical activity, nutrition, and recovery. It emphasizes the importance of maintaining a healthy body to support overall well-being. Mental well-being focuses on cognitive functioning and psychological resilience. This dimension includes aspects such as mental clarity, focus, and the ability to manage stress effectively. Emotional well-being relates to the ability to recognize, understand, and manage emotions. It includes fostering positive emotions, emotional stability, and coping mechanisms for handling challenges. Last, social well-being highlights the importance of interpersonal relationships and social connections. It involves feeling supported, maintaining meaningful relationships, and having a sense of belonging within a community.

This model is particularly relevant when examining the experiences of youth athletes participating in high-performance events like the YOG. The purpose of this study is to explore whether participating in the Youth Olympic Games programs positively influences the well-being of youth elite athletes. By examining how various activities can impact athletes' physical, mental, emotional, and social well-being, this research aims to provide insights into fostering holistic development and enhancing performance in young athletes' well-being in sport.

### Youth elite athletes and the Youth Olympic Games

Understanding youth elite athletes is essential because they represent the future of sports and provide a unique lens through which to study the interplay between athletic performance and developmental growth (24). In particular, the YOG offer a distinctive and valuable forum for such research, bringing together young athletes from diverse backgrounds in a global event that emphasizes both elite competition and holistic development. The International Olympic Committee (IOC) and YOG organizers aim to prioritize youth athletes' well-being, making the YOG an ideal setting for studying how participation in such events shapes young athletes' physical, mental, and social health. The YOG was established in 2010 by the International Olympic Committee (IOC) as an international sporting, cultural, and educational event for athletes aged 15–18 years. Each YOG is designed with a strong emphasis on young athletes' well-being and holistic development, where they could compete, learn, and share experiences, both on and off the field of play, equipping them with skills for their sporting careers and life beyond sport (25).

The YOG concept provides a valuable opportunity to understand more about their contribution to athletes' health and well-being in the context of elite sport. The YOG, designed to help athletes learn about challenges, adopt healthy living habits, and share these experiences with their peers, encourages young athletes to better understand their bodies' needs and limits, thereby minimizing health risks.^1^ Participating when aged 15–18 years, YOG athletes are in a critical stage of mental and emotional development, characterized by heightened receptivity to learning and personal growth (19, 26, 27). This developmental stage amplifies the significance of the YOG as a platform for fostering well-being and resilience among youth elite athletes. However, the elite nature of this event subjects young athletes to significant physical and mental pressures (9, 19, 28–30, 65), which can influence their well-being during this formative stage. The YOG provide opportunities for young athletes to grow through sports participation and educational activities aimed at fostering both athletic skills and personal development (5, 23, 31, 32).

Since the inception of the YOG, studies have demonstrated that participation in this event impacts various dimensions of young athletes' lives, including their athletic and social development (5, 23, 30, 32–35). The IOC and researchers (19) have called for more scientific studies that specifically address the sports well-being of youth and adolescents within the context of their developmental stage in youth elite Olympic sports, including the YOG.

### Understanding youth athletes' well-being through the Giles et al. well-being model

The Giles et al. (11) model of well-being aligns well with the YOG concept. Physical well-being plays a vital role in the overall health and performance of youth athletes, especially young competitors. Sports training provides well-documented physical and psychological benefits that positively impact athletes' well-being (4, 11, 36). However, physical well-being can suffer due to illness, injury, overtraining, poor nutrition, or substance abuse (37, 38). After the first YOG in 2010, Steffen and Engebretsen (39) highlighted the importance of monitoring the physical strain on young athletes at the YOG. Similar concerns were raised in studies by Brito et al. (40), Chia et al. (41), and Ruedl et al. (42) on the negative effects on young athletes' health. Palmer et al. (16) and Tripplet et al. (43) have continued to monitor these young athletes. However, while there is substantial evidence from injury-prevention studies involving youth sports participants (44), this remains an important area of focus as new generations of young athletes and new sports enter the elite sport context of the YOG.

Mental well-being is a critical component of overall health for young athletes, especially in high-pressure competitive environments (8, 45). Participation in youth sports such as the YOG can potentially help athletes develop psychological skills that benefit both their performance and overall well-being (46). However, the competitive nature of the YOG can be stressful (19, 23, 30).

Social well-being is conceptualized by Giles et al. (11) through factors such as positive relationships, social acceptance, coherence, contribution, growth, and integration. While MacIntosh et al. (30) note that the main focus of YOG athletes is performance, they also highlight the importance of social interaction, including national and international friendships among athletes. The YOG can help athletes build networks that enhance both their sports skills and their contributions to their communities (25, 31).

**Figure 1:**
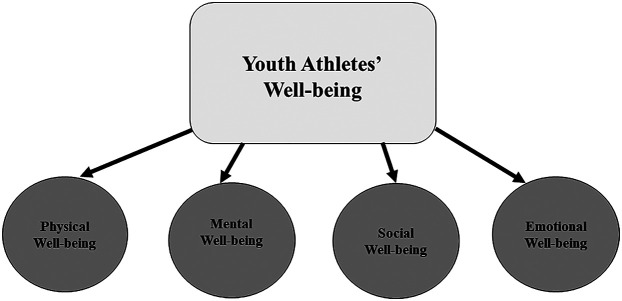
The well-being model for youth athletes`, as adapted from Giles et al. (11).

Emotional well-being, although related to mental health, is considered separately in this study. Feeling good about one's life is a key component of a healthy life and is often described as emotional well-being (21). Giles et al. (11) define emotional well-being through emotional stability, positive and negative emotions, happiness, and life satisfaction. Major sporting events like YOG can provide a sense of accomplishment and build positive character traits in young athletes. This study was therefore designed to advance understanding of how the four aspects of well-being identified by Giles et al. (11), i.e., physical, mental, social, and emotional, apply to YOG athletes (see Figure 1) with the following main research question:

In what ways do youth athletes perceive their participation in the YOG as influencing their physical, mental, social, and emotional well-being?

## Methods

A retrospective mixed-methods approach was used to explore how participation in the Youth Olympic Games (YOG) has influenced athletes' well-being, with a survey of former YOG athletes in late 2017 and follow-up interviews in 2018. In this retrospective design, YOG participants aged 18–26 years at the time of data collection reflected on their past experiences 1–7 years after participating in their respective YOG event. This method has been criticized for potential issues such as “memory, impression, and attributional bias” (47). However, the YOG, as a once-in-a-lifetime event, is considered highly memorable (48). Research shows that our memory enables us to reflect on the past and make informed decisions based on prior experiences (49, 50).

After ethical approval from the University of Sydney Human Research Ethics Committee (approval number 2017/133), invitations were sent to 247 YOG athletes who had taken part in one of the first four YOGs: Singapore 2010 (summer), Innsbruck 2012 (winter), Nanjing 2014 (summer) or Lillehammer 2016 (winter).

### Sample and procedure

The population in this study is YOG athletes from both individual and team YOG sports from two countries: Norway and Singapore. The two chosen countries have both been hosts for a YOG, with Singapore hosting summer sports in 2010 and Norway hosting winter sports in 2016. Both have relatively small populations. The selection of athletes from these specific countries was influenced by the lead researcher's ties to Olympic sports and athletes in these regions. To recruit participants, athletes were contacted up to three times over 16 weeks (October–December 2017), providing a hyperlink to an anonymous survey created using the Research Electronic Data Capture (REDCap) program, which included a built-in consent form.

Of the 191 former athletes who responded, 173 remained after data cleaning, with 82 females (47.4%) and 91 males (52.6%), who were, on average, 16.6 ± 1.0 years old when participating in the YOG. Of the 173 participants, 80 (46.2%) participated in Singapore 2010, 23 (13.3%) in Innsbruck 2012, 39 (22.6%) in Nanjing 2014, and 31 (17.9%) in Lillehammer 2016.

The recruitment process for the interviews was conducted through an online questionnaire linked to a new platform in REDCap, ensuring that responses could not be traced back to the survey. The lead researcher successfully established contact with 50 of the 153 participants who had willingly agreed to the interview and provided their contact details. The 30 interviews were conducted from August to November 2018, approximately 6–8 months after the completion of the survey, either face-to-face in person or online through Skype and/or WhatsApp (mobile application). The sample size of 30 represented one in six survey responders and allowed for more in-depth interviews with each athlete. Of the 30 participants interviewed, 9 were from Singapore 2010, 3 from Innsbruck 2012, 10 from Nanjing 2014, and 8 from Lillehammer 2016. The interview setup allowed the researcher to identify respondents and observe their nonverbal expressions (67). Before each interview, consent was obtained, and the sessions, lasting approximately 20–30 min, were recorded.

### Instruments

The online survey had two parts: the first part asked demographic information, including which YOG the participants had attended, while the second part focused on how participation in the YOG influenced various aspects of their well-being (see Table 1). The survey was developed using insights from the official Olympic Games Research Department's survey distributed to athletes by the IOC,^2^ and reports from the organizing committees (e.g. (51, 52), and other relevant YOG program documents (e.g. 53, 54). Four aspects of well-being were studied with a total of 18 items: items 1–5 on physical well-being, items 6–10 on mental well-being, items 11–14 on social well-being, and items 15–18 on emotional well-being (see Table 1). Participants rated each item on a 5-point Likert scale, ranging from 1 = “fully disagree” to 5 = “fully agree”. Based on Giles et al. (11), the self-report method is appropriate for measuring well-being, as it captures a snapshot of athletes' experiences while also being a practical and cost-effective data collection approach.

**Table 1:** Survey items on four aspects of well-being in the Youth Olympic Games (YOG).

Aspect, item number and statement		Median	Q1–Q3	*P* and *C-*α values
Physical well-being				
1.	I am aware of tools I can use to prevent injuries in my sport (*n* = 168)	4.0	4.0–5.0	<0.001
2.	I learned the importance of staying physically active in everyday life (*n* = 168)	4.0	4.0–5.0	<0.001
3.	I have discovered the importance of motor skills on my sports performance (*n* = 167)	4.0	3.0–5.0	<0.001
4.	I learned the importance of nutrition to perform in my sport from YOG (*n* = 168)	4.0	3.0–5.0	<0.001
5.	I learned about the anti-doping program in YOG (*n* = 168)	4.0	4.0–5.0	<0.001
*The aggregated grand median and IQR, and C-*α *value for physical well-being*		4.0	4.0–5.0	*C-*α = 0.75
Mental well-being				
6.	I started to use mental training in my sport after YOG (*n* = 168)	4.0	3.0–5.0	<0.001
7.	I added more mental training to my program after YOG (*n* = 168)	4.0	3.0–4.0	<0.001
8.	Mental training has helped me in my sports performance (*n* = 168)	4.0	4.0–5.0	<0.001
9.	Mental training has helped me in my daily life (*n* = 167)	4.0	4.0–5.0	<0.001
*The aggregated grand median and IQR, and C-*α *value for mental well-being*		4.0	4.0–5.0	*C-*α = 0.84
Social well-being				
10.	I use social media to engage and inform followers in my sport (*n* = 165)	4.0	3.0–4.0	<0.001
11.	I learned the importance of using my social network to perform better at sport (*n* = 172)	4.0	3.0–4.0	<0.001
12.	I am still in contact with athletes from other cultures I met during YOG (*n* = 173)	4.0	3.0–5.0	<0.001
13.	I have taken on a role in my community (*n* = 164)	3.0	2.0–4.0	0.383
14.	I have taken on a role in my sports club (*n* = 164)	3.5	3.0–4.0	<0.001
*The aggregated grand median and IQR, and C-*α *value for social well-being*		4.0	3.0–4.0	*C-*α = 0.63
Emotional well-being				
15.	YOG gave me the awareness to better express myself through channels other than social media e.g., art, music, and dance (*n* = 165)	3.0	3.0–4.0	<0.001
16.	I became more aware of the importance of what I am sharing on social media (*n* = 165)	4.0	3.0–4.0	<0.001
17.	Athlete role models helped me to realize the importance of a sport–life balance (*n* = 168)	4.0	3.0–5.0	<0.001
18.	I developed an awareness of the programs that the IOC has to encourage safe sport. Such programs include sexual harassment & abuse, health & body image, female athletes & health (*n* = 168)	4.0	3.0–4.0	<0.001
*The aggregated grand median and IQR, and C-*α *value for emotional well-being*		4.0	3.0–4.0	*C-*α = 0.75

Items were rated on a 5-point Likert scale: 1 = fully disagree, 2 = somewhat disagree, 3 = neutral, 4 = somewhat agree and 5 = fully agree. The aggregated grand median and inter-quartile range (IQR) and Cronbach's alpha (*C-*α) value for each aspect of well-being were based on *n* = 162 (participants with data on all 18 items). A *P*-value < 0.05 denotes a significant difference compared to the midpoint score of 3 = “neutral”. Q1–Q3, quartile 1 to quartile 3.

The guide questions for the semi-structured interviews were developed from the same material as for the survey. Each interview started with background questions about the participant's nationality, type of sport, the specific YOG they attended, and their memories from those experiences. The interviewer then asked participants to share their perspectives on well-being and a healthy lifestyle from their participation in the YOG. Norwegian participants were interviewed in Norwegian, while Singaporean participants were interviewed in English. Each interview was transcribed and coded by the lead researcher before the next took place.

To minimize any potential bias during transcription, the verbatim texts were initially transcribed in Norwegian before being translated into English. The English translations were then translated back by the lead researcher to Norwegian to ensure that the athletes' original opinions and perspectives were accurately captured.

### Data analysis

Table 1 presents the median and interquartile range (IQR) responses on the 18 well-being items rated on a 5-point Likert scale. A one-sample Wilcoxon signed-rank test was used to compare responses against a “neutral” response of 3.0 on the Likert scale. For each aspect of well-being, the aggregated grand median and interquartile range (IQR) were calculated by first computing individual median scores across items and then deriving the group median and IQR from these values. Internal consistency of the items within each of the four well-being dimensions was evaluated using Cronbach's alpha. As outlined by Hair et al. (55), factor loadings of 0.5 or higher are deemed acceptable, while values of 0.7 or above are considered ideal. The aggregated grand median values and Cronbach's alpha were calculated using the sample of 162 participants who had complete data for all 18 items of well-being. Sex-group–specific aggregated grand medians for each aspect of well-being (females: *n* = 76; males: *n* = 86) were compared using the Mann–Whitney test, i.e., comparing female vs. male ratings for each of the four dimensions of well-being. The survey data was analyzed using the Statistical Package for Social Sciences (SPSS 29, IBM Corp., Armonk, NY, USA).

The interview data was analyzed using a top-down thematic approach (56) using the qualitative data analysis program NVivo 14 (Lumivero LLC, Denver, CO, USA). Deductive codes based on the theoretical framework of well-being in athletes (e.g., physical, mental, social, and emotional well-being) were used. The node-matrix in NVivo contained specific references to sections of the transcripts related to key themes such as injury prevention knowledge, nutrition, doping, mental training, support systems, coaches, athlete role models, psychological support, physiological care, mental impact, social life, belonging, staying physically active, health in life, and sport–life balance. To enhance the validity and accuracy of the findings, peer reviews (57) were conducted to identify relationships among the data and to group them into the four themes aligned with the well-being aspects proposed by Giles et al. (11). The 18 items were tested using principal components analysis, and they supported/confirmed the 4 well-being elements that Giles et al. (11) later published.

## Results and discussion

Table 1 presents the quantitative results for the four dimensions of well-being: physical, mental, social, and emotional. These findings are supplemented by qualitative data from interviews, exploring each well-being dimension.

Table 1 shows that internal consistency was relatively acceptable for all four dimensions (Cronbach's *α* = 0.63–0.84) as only one category, social well-being, fell slightly below the 0.7 threshold. When aggregated, the grand medians indicated positive effects across all dimensions, with values consistently above the neutral midpoint (i.e., above 3 on the 1–5 scale). Physical well-being and mental well-being were the most impacted by YOG participation, aligning with previous studies that emphasize physical well-being as the most examined dimension (1, 4) and that injury prevention tends to be the most impacted and studied direction of YOG athletes' health (39, 40, 68). Female and male ratings were similar for physical well-being [both: median of 4.0 (IQR: 4.0–5.0), *P* = 0.304], mental well-being [both: median of 4.0 (IQR: 3.5–5.0), *P* = 0.883], and social well-being [both: median of 4.0 (IQR: 3.0–4.0), *P* = 0.454]. However, a small sex difference emerged for emotional well-being, with males reporting slightly higher values [males: median of 4.0 (IQR: 3.5–4.5); females: median of 3.5 (IQR: 3.0–4.0), *P* = 0.009]. The following paragraphs summarize the most critical subthemes for each well-being dimension that emerged from the survey and interview responses.

### Influence of the YOG on physical well-being

Physical well-being is one of the four core aspects of athletes' well-being in the Giles et al. (11) model. In the survey and semi-structured interviews, athletes reflected on the physical aspect of well-being. In the survey, physical well-being was measured in five items, such as “I learned the importance of staying physically active in everyday life”. Most respondents agreed that the YOG helped them develop an awareness of injury prevention tools and awareness of the IOC anti-doping program. This aligns with the IOC's^1^ goal of promoting a better understanding of athletes' physical needs to minimize health risks. It also supports existing literature (66) that emphasizes the role of injury prevention in improving youth athletes' health. Anti-doping, an emerging area in youth sport research, was positively impacted by YOG participation, supporting earlier studies by Mountjoy et al. (58) and Krieger and Kristiansen (59).

To explore these survey findings, qualitative data collected on physical well-being reinforced the importance of injury prevention, which aligns with the work of Schnitzer et al. (35) on YOG athletes in Innsbruck 2012. The Palmer et al. (16) study complements the above findings, showing that while the rate and characteristics of injury and illness varied between sports, consistent risk prevention strategies in all areas could have a positive impact on the health and well-being of YOG athletes. Injury prevention tools were viewed as essential for athletes aiming to sustain their sports careers, helping them manage training loads and avoid career-ending injuries. For some athletes, even after stepping away from competitive sports, these tools remained valuable. One athlete from Lillehammer 2016 shared lessons learned and the impact, also found in the statements of other athletes.

I went to injury prevention activities and there I learned useful exercises I practice today through strength training to prevent injuries. I still use them today even if I am not as active as I used to be. N10 (Lillehammer 2016 YOG)

Athletes reported learning to firmly reject doping, reflecting the success of the IOC's anti-doping goals, as also noted by Mountjoy et al. (58). Moreover, Backhouse et al. (37) explored the best anti-doping strategies for youth athletes and emphasized the critical role of socialisation and significant others in amplifying and dampening the impact of doping within youth sport. In discussing anti-doping, many athletes also highlighted the importance of a healthy life in sport, attitudes in general, and a particular example of cardiopulmonary resuscitation as a critical skill for athletes. One athlete stated:

I did the WADA doping booth, and the other one was the CPR [cardiopulmonary resuscitation] that was interesting. I think those were skills I took with me that are important to know about in sport. S7 (Nanjing 2014 YOG)

It is essential to extend anti-doping education beyond the YOG to other youth sports events. This aligns with the recommendations of Backhouse et al. (37), who emphasized the importance of reinforcing doping prevention efforts in youth sports. Gatterer et al. (60) built on that and specified that while all anti-doping education is paramount for prevention, action-based programs are more effective than “information-use” only. As the youth athletes reflected on the impact of YOG and the importance of physical well-being, the third theme of nutrition emerged. Many athletes reported positive experiences from nutrition classes at the YOG, although some felt that it was acceptable to eat less healthy foods occasionally, despite the need for adequate nutrition to support their hard training schedules. Overall, the topic of sports nutrition captured the athletes' attention, as illustrated by the following statements:

In YOG we had cooking classes and guidance on how we should put together a healthy diet. N3 (Lillehammer 2016 YOG)

During the cooking class they taught me certain things, regarding what to use in ingredients as in getting enough of everything. S2 (Nanjing 2014 YOG)

Desbrow (61) supports the development of healthy nutritional habits at young ages, as young athletes are not only undergoing significant physical growth and increased energy demands but also establishing a foundation for lifelong nutritional understanding. Desbrow (61) and Bergeron et al. (19) emphasised the health and performance advantage of a nourishing, balanced diet with ample energy, and that healthy nutrition needs to be managed and reinforced without misguided pressure and persuasion to achieve premature muscle development. The IOC (22) recognizes the importance of this topic, making the YOG an ideal platform to promote nutritional awareness. In the long term, clear messaging on nutrition can enhance athletes' physical quality of life.

**Figure 2:**
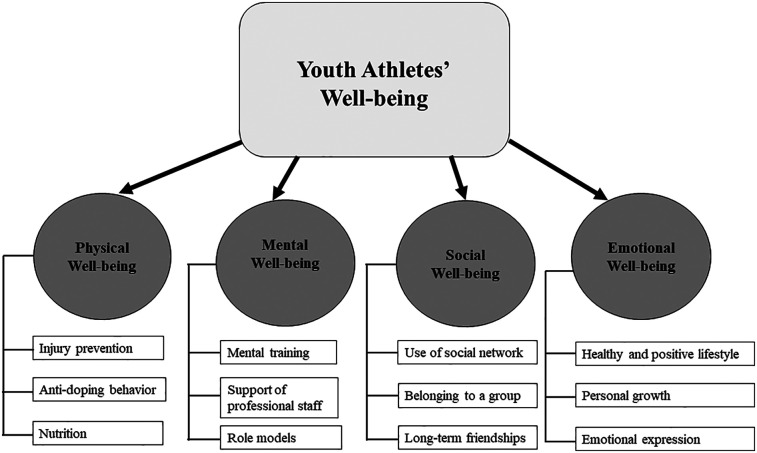
An extended well-being model for youth athletes'.

In total, based on the survey and interview results, YOG participation appears to positively influence youth athletes' physical well-being, with particular emphasis on injury prevention, anti-doping values, and the importance of nutrition [see the extended Giles et al. (11) model in Figure 2].

### Influence of the YOG on mental well-being

Mental well-being was measured with four survey items, such as “Mental training has helped me in my sports performance”. The median scores of 4.0 (i.e., *“*somewhat agree”) for each of the items show that the YOG athletes agreed that their participation in the YOG notably positively improved their mental well-being. The survey results were complemented by the interview data with questions on mental well-being. Many athletes articulated their realization of the importance of mental resilience for athletic performance gained through YOG activities with instructors or through competition itself. One Norwegian athlete reflected on the enduring impact of mental training facilitated by a mental coach, highlighting the importance of adequate sleep and its effects on focus by stating:

Talk on mental training by a mental coach on how to maximise your performance. How important it is to sleep enough, and how strong an effect sleep has on staying focused and how this affects long and short-term focus. N6 (Lillehammer 2016 YOG)

This finding is consistent with literature indicating that many YOG athletes progress to compete at higher levels, where mental training is essential for coping with pressure and avoiding performance anxiety (36, 45). Implementing mental training at a young age can foster positive competitive well-being and help youth manage anxiety (24, 45). Another athlete illustrated how the mental strength developed through the YOG competition helped the athlete to manage pressure beyond sports:

So, when I am in a bit of pressured situations, I always think of my finale in YOG in Nanjing. This gets me very focused on the tasks I have to do so to forget to be nervous. I guess this is the strongest moment I have taken away with me mentally from the YOG. I used this episode last time just before I did my driving license and all went well, and I was not really nervous. N2 (Nanjing 2014 YOG)

Athletes also noted the impact of role model support networks during the YOG event, as interactions with the former Olympians provided strategies for managing competitive pressure. Such a support network is crucial for helping athletes develop coping mechanisms and enhance their overall experience in sports (23, 62). One athlete shared an inspiring technique from a role model, who used imaginative scenarios for self-motivation:

[Role model]… shared with me how I can prepare myself mentally and what I could think of coming into positions… she told me she used to imagine she was an ice queen with strong straight back high head… she did this to get a strong belief in herself. In the track, she had a bear that was hunting her… all this I have taken with me. N7 (Lillehammer 2016 YOG)

Participating in the YOG provides young athletes with unique opportunities to interact with athlete role models (23), allowing them to learn and develop personal mental imagery techniques to strengthen their mental resilience. Based on survey and interview results, YOG participation is likely to positively influence youth athletes' mental well-being, particularly emphasizing the significance of mental training, support from professional staff, and the influence of athlete role models, These concepts are demonstrated in Figure 2.

### Influence of the YOG on social well-being

Social well-being is one of the most narrowly studied aspects of well-being, with a dominant focus on the positive relationships component and a lack of attention to other potential components, such as social acceptance, social contribution, and social integration (11).

In the survey and interviews, athletes reflected on the social aspect of well-being. The survey measured social well-being with five items, such as “I learned the importance of using my social network to perform better at sport”. Most participants reported a relatively lower impact of participating in the YOG on their social well-being compared to other aspects of their well-being. One item with high response in social well-being impact was the importance of using social networks to enhance sports performance. This was reinforced in the interviews, where participants emphasized the lifelong value of building networks and friendships in sports. One athlete, for example, highlighted the long-term importance of such relationships:

The YOG gives you a platform to interact with other athletes from other nations and get to know them better. It helps you to build a network and I think it will be helpful in the future as an athlete if one wants to train overseas and sort out the logistics and help each other out as we do in the sport I come from. Those relationships stay with you for life. S11 (Nanjing 2014 YOG)

This supports the findings of Macintosh et al. (30), who argued that one of the primary contributions of the YOG is fostering performance-focused social interaction among athletes, which helps build networks to improve sporting performance.

Another key theme that emerged from the interviews was the importance of a high-quality social life and a sense of belonging to a group. For instance, one Norwegian athlete stressed the role of social interaction in their overall well-being, identifying it as a highly positive aspect of the YOG experience:

It is important to have a milieu to train in. To me well-being is being social and being together as a group, and this was what made YOG a strong well-being for me. N10 (Lillehammer 2016 YOG)

Another important element of social well-being reported by YOG athletes was forming long-term friendships. Many mentioned that the YOG encouraged them to form friendships, which promoted a sense of belonging in sport and provided a social network post-games. This aligns with Parent et al. (34), who emphasized the value of friendship in sports and highlighted the YOG's role as a valuable platform for fostering these relationships. MacIntosh et al. (30) also recognized this, seeing the holistic development of young athletes, both on and off the field, as a central aim of the YOG. The long-lasting effects of these friendships were evident in the experiences of athletes who continued to maintain connections with fellow competitors from around the world. One athlete also reflected on the social and cultural exchanges and enduring friendships formed during the YOG:

The best with YOG was the cultural aspect, and we get the chance to get to know well other athletes and their culture. For myself I had never heard about the Virgin Islands before I came to YOG, and we are still in contact today. The program itself had a strong emphasis on getting to know each other from other countries and cultures. I guess what I took away from YOG was the friendship and friends I made, which I wouldn't have if I didn't participate in YOG. Even up to today, 8 years after, I still have contact with several of the athletes I met during the YOG! I do think we get forced in a positive way to make contact and communicate with each other, since we are only one from each nation in many sports. This I think was and still is great because thanks to the YOG I have built an international network I still have contact with. N5 (Singapore 2010 YOG)

In summary, YOG participation had a positive impact on youth athletes' social well-being, particularly three elements: building networks within sport, experiencing a strong sense of belonging, and building long-term friendships. These experiences contribute to the athletes' long-term social well-being and development, both in sport and beyond.

### Influence of the YOG on emotional well-being

In the survey, emotional well-being was measured in four items, such as “YOG gave me the awareness to better express myself through channels like art, music, dance”. Most participants reported a relatively high impact of participating in the YOG on the emotional aspect of their well-being compared to other aspects of their well-being. The qualitative findings enrich our understanding of emotional well-being. In particular, the interviews reveal how athletes learned to balance sport and life through interactions with athlete role models, as expressed by this athlete:

Education is important because sport is not going to last forever. You can only do sport to 30+ professionally, and then you still need to go out and work. So this was stressed from the athlete role models, but the reason for me to study is different. It was my sport that inspired my field of study, so I am taking a degree outside sport to become a researcher in my field. The second reason is because it offers an outlet for me to channel my energy outside my sport, so I am not overthinking my sport. I kind of separate my sport and everything else. So, school helps me with that, but I am lucky I like my field at university. S7 (Nanjing 2014 YOG)

This finding aligns with Li and Powdthavee (69), who emphasized that additional education could raise awareness about maintaining a healthier lifestyle after a sports career ends, and with Staalstroem (7)), who highlighted the importance of addressing young athletes' holistic development.

Another theme that emerged from the qualitative analysis was the importance of maintaining a positive lifestyle, ranging from the joy of participation to the disappointment of defeat. The participants reflected on the significance of emotional well-being, with several citing the YOG as a source of influence. One athlete described their experience of activities with the athlete role models on staying active and positive, even during challenging times:

I went to a talk on healthy lifestyle on how to live a healthy life, stay positive and active even on days that don't go as well or the challenging days. N10 (Lillehammer 2016 YOG)

In promoting emotional health, the YOG played an essential role in fostering social interactions and personal growth. Athletes described how the event encouraged them to engage with others, build relationships, and become more open and mature in social settings. One athlete shared how the YOG helped them break out of their shell:

What I took with me was to dare to bond and build relations, to be myself and dare to talk to all the other athletes. YOG helped me to open up more and talk to other athletes, and just be more open in general. I still have contact with several athletes from other countries, and to have contact and know other athletes from other countries is really enjoyable. N13 (Nanjing 2014 YOG)

The responses also showed that emotional well-being is strongly tied to the athletes' sense of personal growth and achievement and continued passion for their sport. One athlete expressed the fulfillment of achieving their dream of competing at the Olympics:

I also took part in the Olympics, and you know when you dream of something and suddenly one is there, it is just a different feeling all together. I am so glad I worked hard to manage to get to the YOG, Olympic level and manage an education as well, and the best is I still love my sport even though I am not really doing it full time at the moment. S10 (Singapore 2010 YOG)

Another athlete similarly expressed how the YOG had a lasting impact on their emotional skills and emotional expression development:

The one thing I took with me from YOG that I still use today…mmmm all the things I can think of are being more open and talking to people. I used to be so introverted, but after YOG I changed and am more daring to talk to people, be more sociable and outgoing in the sense that before I went there, I didn't talk to any other athletes when I went overseas, or in local events I didn't communicate or interact with them. But after being through the whole YOG I became more open so when I now go to other competitions, I am more open. I actually try to say hi or I actually talk to them now. S12 (Nanjing 2014 YOG)

Again, the findings support that it is important for the athletes to build friendships (34, 63) to be part of a community. The findings also support that youth athletes build awareness of the importance of education while being in the YOG (31). These aspects will influence emotion that can range from the joy of being social, belonging to a sports group through participation, and having education to turn to when the disappointment of defeat hits. In summary, the survey and interview results indicate that YOG participation positively impacted youth athletes' emotional well-being, with a key focus on maintaining a healthy and positive lifestyle, personal growth, and emotional expression.

Figure 2 summarizes the key impacts on the well-being of youth athletes based on an adjusted and extended Giles et al. (11) well-being model. The extended model depicts the four original aspects of youth well-being and the three extra components for each produced by our study.

This study aimed to develop an understanding of how YOG participation influences athletes' well-being. While the findings suggest that YOG can positively affect youth athletes in multiple ways, it is also crucial that organizers of sports events take young athletes' well-being seriously. This includes providing strong support to ensure that the event promotes holistic health, not just competitive achievement.

### Theoretical and practical implications

Our study concludes with several key findings and identifies several key implications that the IOC could find helpful and important to review and implement to strengthen the impact of YOG participation on youth athletes' well-being. One of the main theoretical contributions of our study is the extension of the Giles et al. (11) framework to the YOG context. We tested the four dimensions of well-being in the understudied setting of young elite athletes and extended the Giles et al. (11) model. Our results indicate that all four aspects of well-being were likely to be positively influenced by YOG participation, but social well-being had a somewhat lower impact, although still positive.

On a practical level, the findings can help YOG organizers and practitioners understand the importance of fostering all four dimensions of well-being. Athletes and their coaches should be provided with clear frameworks and guidelines to address each dimension. For physical well-being, the study showed that YOG participation had positive impacts, particularly in injury prevention, anti-doping values, and the importance of nutrition. For mental well-being, the study showed the value of mental training, professional support, and the influence of athlete role models. For social well-being, the study showed that YOG participation contributed to an improved quality of social networks, to a sense of belonging within a group, and to the development of long-term friendships. Lastly, for emotional well-being, the study showed YOG participation contributed to maintaining a healthy and positive lifestyle, to personal growth, and to emotional expression.

Our study suggests that more attention should be given to developing tools and strategies that support athletes' social and emotional well-being in the YOG context. This can be achieved through a combination of sports, cultural interactions, involvement with athlete role models before and after the games, and activities in the educational programs, which together can provide a more holistic experience for the youth athletes. By adopting a comprehensive approach that fosters all dimensions of well-being, YOG participation can have lasting benefits for youth athletes, both during the games and throughout their future careers.

## Limitations

Although our study yielded valuable insights, it is important to highlight its limitations. First, the sample size of 173 athletes, with athletes from only two countries, limits the generalizability of our findings. Expanding the sample to include a broader representation of YOG athletes would be beneficial for future research. Second, interviews and data collection took place 1–7 years after the respective YOG events, allowing athletes time to reflect on the past (49, 50) and the impact of this significant life experience (64). However, this retrospective approach could also be seen as a limitation, as it may affect the accuracy of memory recall (47). Third, our study focused exclusively on the perspectives of YOG athletes. In line with the recommendations of Bennie et al. (70), incorporating the views of coaches, psychologists, and team managers from the national teams in the post-YOG period would provide a more comprehensive understanding. These adults are well-positioned to assess the long-term impact of YOG participation on young athletes. Fourth, the instruments used in this study were designed before the Giles et al. (11) model came out, and do not measure psychometric dimensions but activities that can support these dimensions. Fifth, although the study included participants who competed in a wide range of sports, 13 summer and 7 winter sports with both team and individual sports from the approximately 28 summer and 7 winter sports in the 2010–2016 YOGs, the study did not collect data on the specific sport of each participant. Exploring the impacts of particular sports, or the individual vs. team sport perspective, is another research direction for YOG and well-being research. Finally, we recognize that this study did not include a comprehensive assessment of well-being across a range of scales.

## Conclusion

This study investigated the influence of participation in the YOG on four key dimensions of well-being among young elite athletes: physical, mental, social and emotional well-being. The quantitative findings demonstrated significant positive effects across all dimensions, with physical and mental well-being being most strongly influenced. These outcomes were corroborated by qualitative analyses, which provided deeper insights into the mechanisms underpinning these effects.

Specifically, YOG participation was found to enhance physical well-being through increased awareness of injury prevention, anti-doping practices, and nutrition. Mental well-being was strengthened by exposure to mental training, professional staff support, and role models. Social well-being was fostered by social networks, a sense of group belonging, and the development of long-term relationships. Emotional well-being was positively influenced by the promotion of healthy lifestyle choices, opportunities for personal growth, and avenues for emotional expression.

Overall, the findings suggest that participation in the YOG promotes the development of healthier, more knowledgeable youth athletes and contributes to their broader social integration. To maximize these benefits, the well-being of YOG athletes should be addressed holistically, recognizing the interdependence of physical, mental, social, and emotional health. Investment in health education and safe sport practices not only supports athletic performance but also facilitates the personal development of youth athletes as responsible and positive members of society.

## Data Availability

The raw data supporting the conclusions of this article will be made available by the authors, without undue reservation.

## Footnotes

^1^IOC. *Youth Olympic Games Event Manual*. 7th ed. Lausanne: Olympic Study Centre (2015). (Unpublished material).

^2^IOC. *Lillehammer 2016-YOG Post-Games Survey Report*. Lausanne: Olympic Study Centre, Olympic Games Department (2016). (Unpublished material).
